# Decreased grey matter volumes in unaffected mothers of individuals with autism spectrum disorder reflect the broader autism endophenotype

**DOI:** 10.1038/s41598-021-89393-z

**Published:** 2021-05-11

**Authors:** Kyung-min An, Takashi Ikeda, Tetsu Hirosawa, Ken Yaoi, Yuko Yoshimura, Chiaki Hasegawa, Sanae Tanaka, Daisuke N. Saito, Mitsuru Kikuchi

**Affiliations:** 1 Research Center for Child Mental Development, Kanazawa University, 13-1 Takara-machi, Kanazawa, 920-8640 Japan; 2 Division of Socio-Cognitive-Neuroscience, Department of Child Development, United Graduate School of Child Development, Osaka University, Kanazawa University, Hamamatsu University School of Medicine, Chiba University, and University of Fukui, Kanazawa, Japan; 3 Institute of Human and Social Sciences, Kanazawa University, Kanazawa, Japan; 4 Department of Psychiatry and Behavioral Science, Kanazawa University, Kanazawa, Japan

**Keywords:** Neuroscience, Anatomy

## Abstract

Autism spectrum disorder (ASD) is a neurodevelopmental disorder with an early onset and a strong genetic origin. Unaffected relatives may present similar but subthreshold characteristics of ASD. This broader autism phenotype is especially prevalent in the parents of individuals with ASD, suggesting that it has heritable factors. Although previous studies have demonstrated brain morphometry differences in ASD, they are poorly understood in parents of individuals with ASD. Here, we estimated grey matter volume in 45 mothers of children with ASD (mASD) and 46 age-, sex-, and handedness-matched controls using whole-brain voxel-based morphometry analysis. The mASD group had smaller grey matter volume in the right middle temporal gyrus, temporoparietal junction, cerebellum, and parahippocampal gyrus compared with the control group. Furthermore, we analysed the correlations of these brain volumes with ASD behavioural characteristics using autism spectrum quotient (AQ) and systemizing quotient (SQ) scores, which measure general autistic traits and the drive to systemize. Smaller volumes in the middle temporal gyrus and temporoparietal junction correlated with higher SQ scores, and smaller volumes in the cerebellum and parahippocampal gyrus correlated with higher AQ scores. Our findings suggest that atypical grey matter volumes in mASD may represent one of the neurostructural endophenotypes of ASD.

## Introduction

Autism spectrum disorder (ASD) is an early-onset neurodevelopmental disorder that is characterized by social and communication deficits, restricted interests, and repetitive behaviours^1^. ASD may develop as a result of complex interactions between genetic vulnerability and environmental factors during critical neurodevelopmental periods^2^. Although the exact cause of ASD remains unclear, ASD is one of the most heritable major neurodevelopmental disorders^3,4^. A recent meta-analysis reported its heritability as between 61 and 91%, suggesting that ASD has a substantial genetic component^5^.

According to genetic epidemiological data, relatives of individuals with ASD express subclinical autism-like personality traits^6,7^. When Kanner and Asperger described autism, they reported that parents of individuals with ASD have autistic traits, such as mild obsessiveness, late speech onset, and problems relating to the outside world^8,9^. Most previous studies have reported that relatives of individuals with ASD experience more impaired communication, rigid behaviour, mentalizing deficits^10–16^, and general autistic traits^17,18^ than the general population. In relatives of individuals with ASD, autistic traits have been observed not only in behavioural and cognitive aspects, but also in dysregulated neurochemical features, such as whole blood serotonin levels^19^, reelin levels^20^, and amino acid metabolism^21^.

It has been suggested that these types of subthreshold autistic traits are part of the broader autism phenotype (BAP)^22^. BAP traits are more prevalent in first-degree relatives of ASD probands compared with other groups, which supports the assumption of the genetic heritability of ASD^23,24^. By studying the parents of individuals with ASD, it may be possible to further understand the different endophenotypes (also called intermediate phenotypes) of ASD^25,26^. An endophenotype is a heritable and quantitative trait that is intermediary between disease symptoms and the genes associated with the disease^27^.

Regarding brain structure and function in ASD, previous neuroimaging studies have reported that individuals with ASD show abnormalities in many brain regions, including the superior temporal sulcus, middle temporal gyrus (MTG), temporoparietal junction (TPJ), fusiform gyrus, amygdala, anterior cingulate cortex, medial prefrontal cortex, inferior frontal gyrus, hippocampus, parahippocampal gyrus, corpus callosum, and cerebellum^28–34^. Although atypical brain structures in ASD have frequently been reported, the brain structures of parents of individuals with ASD are poorly understood and findings remain controversial^25,26,35,36^. Rojas et al. reported that the parents of individuals with ASD showed greater left hippocampal volume than a control group^36^. Peterson et al. found that the parents of individuals with ASD had increased volumes in some brain areas, including the superior temporal gyri, inferior and middle frontal gyri, superior parietal lobule, and anterior cingulate, as well as decreased volume in the left anterior cerebellar hemisphere^35^. In contrast, Palmen et al. reported no significant differences in brain structure of parents of individuals with ASD compared with a control group^26^. The discrepant findings between previous studies on the parents of individuals with ASD may be the result of different analytical methods (e.g. manual tracing, automatic tracing, or whole-brain voxel-based morphometry [VBM]), subject numbers (which is related to statistical power), or sex distribution of subjects.

In the present study, we applied whole-brain VBM analysis to 45 mothers of children with ASD (mASD) and 46 age-, sex-, and handedness-matched controls. We compared grey matter (GM) volume between the mASD and control groups to investigate the heritable aspects of brain structure and the neuroendophenotypes of ASD. We hypothesized that the mASD group would show some of the atypical brain structure patterns that have been previously reported in individuals with ASD. Furthermore, we aimed to determine whether brain volume abnormalities in mASD are correlated with scores on the autism spectrum quotient (AQ) and the systemizing quotient (SQ), which measure general autistic traits and the drive to systemize^37,38^.

## Methods

### Participants

We recruited 46 healthy mothers of children with ASD (mASD group) and 48 age-matched healthy mothers of typically developing children (control group). Owing to low data quality, we excluded the data of one participant from the mASD group and the data of two participants from the control group. We analysed the data of 45 healthy mothers of children with ASD for the mASD group (mean age = 38.33 years, standard deviation [SD] = 4.33). We confirmed the diagnoses of the children with ASD using the Diagnostic and Statistical Manual of Mental Disorders Fifth Edition (DSM-V) criteria^1^, the Diagnostic Interview for Social and Communication Disorders^39^, and/or the Autism Diagnostic Observational Schedule–Generic^40^. All diagnoses were confirmed by local psychiatrists and clinical speech therapists. We used data from 46 age-matched healthy mothers of typically developing children for the control group (mean age = 38.78 years, SD = 3.98). All participants were recruited from public nursery schools in Kanazawa city, Kanazawa University Hospital, and prefectural hospitals in Toyama. Parents who reported difficulties in daily life because of their own intelligence level, or who were being treated for any mental illness, were excluded from this study. None of the participants had received an official diagnosis of autism. All participants provided full written informed consent to participate in the study, and the procedures were approved by the ethics committee of Kanazawa University Hospital. All methods were performed in accordance with the Kanazawa University Hospital Ethics Committee guidelines and regulations.

The Edinburgh Handedness Inventory^41^ revealed that most participants were right-handed, except for one left-handed and one ambidextrous participant per group. We matched the groups on age, sex, and handedness to reduce potential confounding factors.

To evaluate the autistic-like behavioural features of the two groups, we used the AQ, which measures autistic traits^38^, and the SQ, which measures the drive to systemize^37^. Both assessments are self-report questionnaires. Total AQ scores can range from 0 to 50; a higher AQ score indicates that the individual has more ‘autistic-like’ behaviours. Total SQ scores can range from 0 to 80; a higher SQ score corresponds to a greater drive to systemize and is a behavioural characteristic of ASD. Additional participant details are shown in Table 1.

**Table 1:** Participant characteristics.

Characteristics	Controls (*n* = 46)	mASD (*n* = 45)	*t*	*P*
Age in years	38.78 ± 3.98	38.33 ± 4.33	0.516	0.607
Handedness (right/left/ambidextrous)	44/1/1	43/1/1		
AQ	14.74 ± 6.74	17.31 ± 7.40	− 1.734	0.086
SQ	10.98 ± 5.75	14.60 ± 10.02	− 2.109	0.039
TIV	1396.44 ± 88.17	1355.37 ± 92.19	2.172	0.033*
TGM	619.62 ± 37.97	606.73 ± 37.79	1.623	0.108
TWM	486.82 ± 42.48	464.50 ± 39.75	2.587	0.011*
CSF	289.47 ± 40.04	283.62 ± 49.27	0.623	0.535

Means ± standard deviations and accompanying statistics (two-sided *t*-tests) for participant characteristics. There were no significant differences in age, AQ score, or SQ score between the control and mASD groups (two-sample *t*-tests with FDR multiple corrections). TIVs were significantly different between the control and mASD groups. *P < 0.05.

*AQ* autism spectrum quotient, *mASD* mothers of individuals with autism spectrum disorder, *SQ* systemizing quotient, *TIV* total intracranial volume, *TGM* total grey matter volume, *TWM* total white matter volume, *CSF* cerebrospinal fluid, *FDR* false discovery rate.

### Magnetic resonance imaging (MRI) acquisition

Structural MRI scans were acquired using a 1.5 T MRI scanner (SIGNA Explorer, GE Healthcare, Chicago, IL, USA) with a T1-weighted Fast SPGR sequence using the following parameters: repetition time = 8.364 ms, echo time = 3.424 ms, flip angle = 12°, field of view = 260 mm, matrix size = 512 × 512 pixels, slice thickness = 1 mm, and 176 transaxial images.

### VBM analysis

VBM was performed using the Computational Anatomical Toolbox 12 (CAT12; Structural Brain Mapping Group, Jena University Hospital, Jena, Germany) implemented in Statistical Parametric Mapping 12 (SPM12; Wellcome Trust Centre for Neuroimaging, London, UK).

T1-weighted anatomical images were corrected for bias-field inhomogeneities, and were then segmented into GM, white matter (WM), and cerebrospinal fluid (CSF)^42^. The total GM, WM, and CSF volume was calculated as the total intracranial volume (TIV). Each tissue class was spatially normalized into the DARTEL template in Montreal Neurological Institute space, which is derived from 555 healthy subjects aged between 20 and 80 years from the IXI-database^43^. The segmentation process was further extended by accounting for partial volume estimations^44^. We tested data quality and sample homogeneity using Mahalanobis distance algorithms implemented in CAT12. Mahalanobis distance combines weighted overall image quality, which is a measure of noise and other image artefacts (e.g. motion) before preprocessing, and mean correlation, which is a measure of the data homogeneity after preprocessing. We excluded the data of one participant from the mASD group and the data of two participants from the control group, because the Mahalanobis distance was larger than 2 SDs for these data. For further analysis, we used data from 45 mASD group participants and 46 control group participants. The preprocessed scans were smoothed using a Gaussian kernel of 6 mm (full width at half maximum).

### Statistical analysis

We compared absolute volumes of GM structures using modulated images. To analyse the GM differences between the mASD and control groups, we performed voxel-wise two-sample *t*-tests using the general linear model implemented in SPM12, which uses Gaussian random field theory. We used the covariates of total GM and age as potential confounders in the general linear model.

The clusters were considered significant at *P* < 0.05 after correcting for false discovery rate (FDR) comparisons (with initial peak-level thresholding at *P* < 0.001 and clusters > 200 voxels). Automated anatomical labelling was used to label the significant clusters^45^.

The volumes of the significant clusters were extracted, and Spearman’s rho test was conducted with AQ and SQ scores using the Statistical Package for Social Sciences (SPSS, Version 25). For all statistical analyses, we used an alpha level of 0.05 with FDR multiple corrections.

### Ethics approval and consent to participate

This study was approved by the ethics committee of Kanazawa University Hospital. After receiving a complete explanation of the study, all participants provided full written informed consent.

## Results

For autistic-like behavioural features, there was no significant difference in AQ and SQ scores between the two groups with FDR correction (AQ scores: *t*(89) =  − 1.734, *P* = 0.086; SQ scores: *t*(89) =  − 2.109, *P* = 0.039).

**Figure 1:**
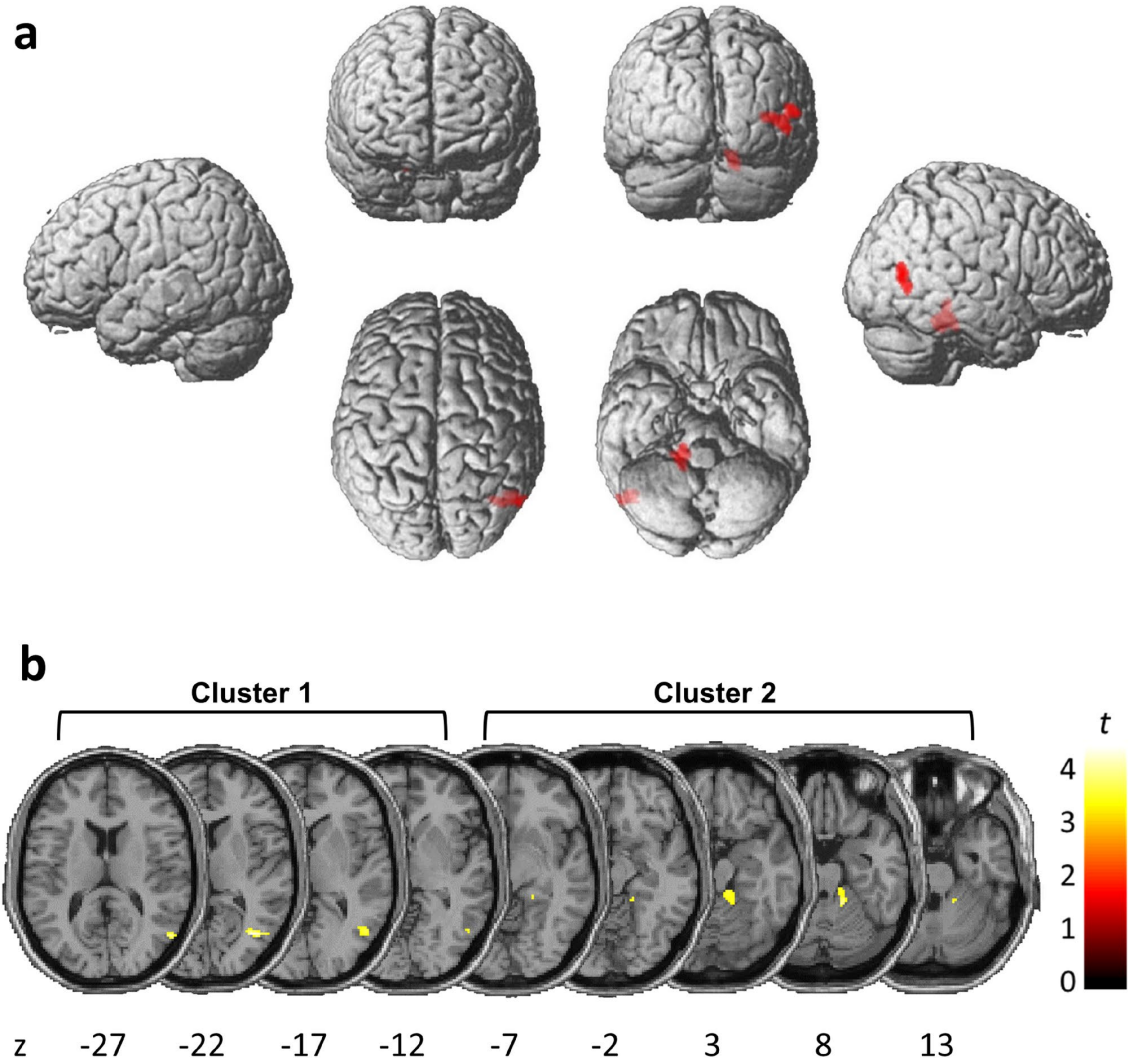
Grey matter volume differences between the mASD and control groups. (**a**) Areas of decreased grey matter volume in the mASD group, compared with the control group, from the voxel-wise two-sample *t*-test. All clusters shown in the results survived thresholding at *P* < 0.05 after FDR correction. (**b**) Two clusters were significantly smaller in the mASD group. Numbers denote MNI coordinates. The colour intensity represents *t*-statistic values at the voxel level. The results are visualized on standard normalized T1-weighted images in selected slices and displayed in accordance with neurological convention (i.e. right hemisphere on the right). *mASD* mothers of individuals with autism spectrum disorder, *FDR* false discovery rate, *MNI* Montreal Neurological Institute.

The VBM analysis demonstrated that GM volumes in several brain regions were smaller in the mASD group relative to the control group. Two clusters showed statistically significant differences between the mASD and control groups (Fig. 1).

**Figure 2:**
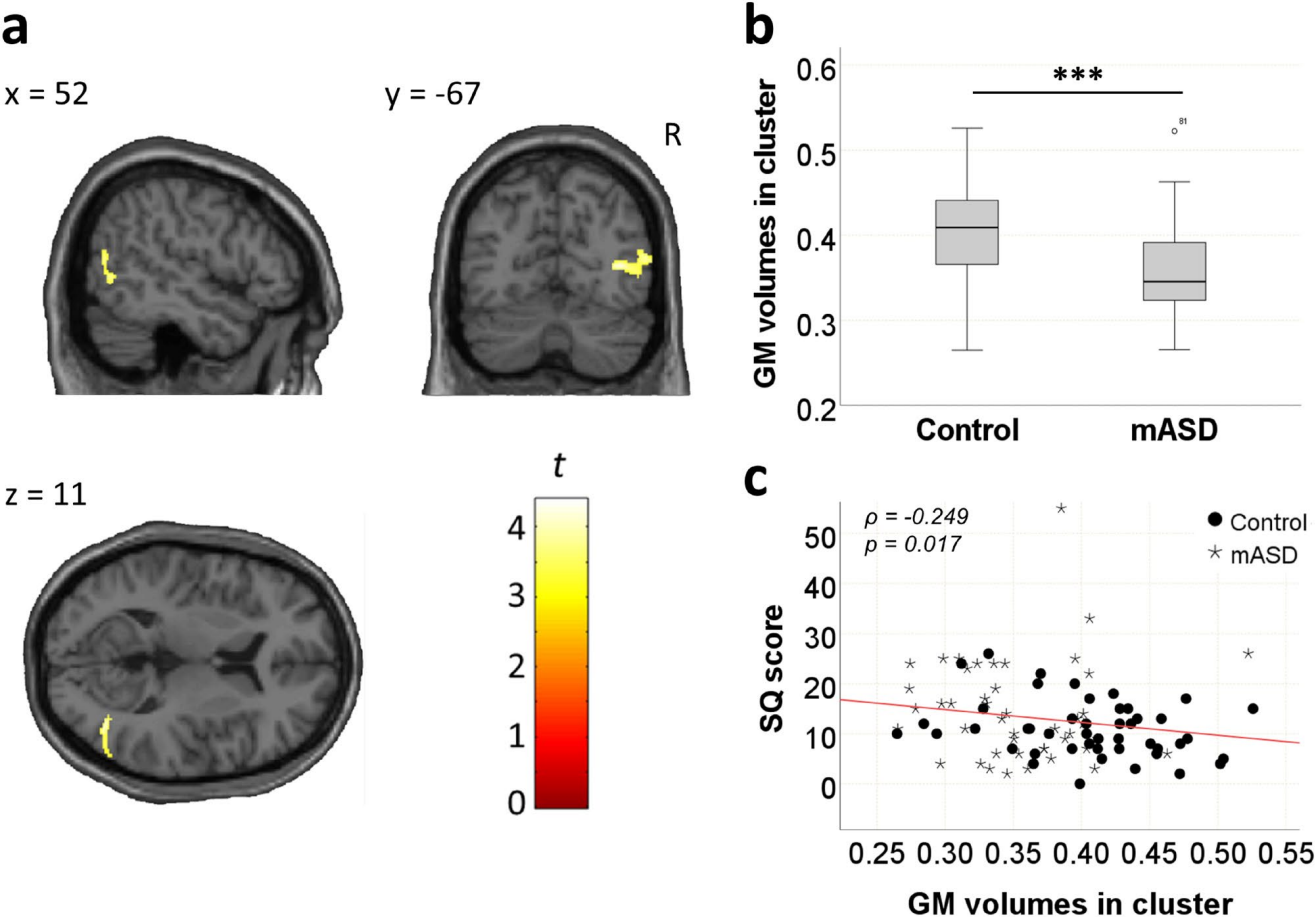
Grey matter volume differences between the mASD and control groups in the first cluster. (**a**) The first significant cluster was observed in the right middle temporal gyrus. (**b**) Grey matter volume of the cluster was significantly different between the mASD and control groups (*t*(89) = 4.330*, P* = 0.000). (**c**) Scatter plot showing negative correlation between the grey matter extraction of the cluster and SQ scores for all subjects. The grey matter volume of the cluster was negatively correlated with SQ scores (ρ =  − 0.249, *P* = 0.017). ***P < 0.001. *GM* grey matter, *mASD* mothers of individuals with autism spectrum disorder, *SQ* systemizing quotient.

**Figure 3:**
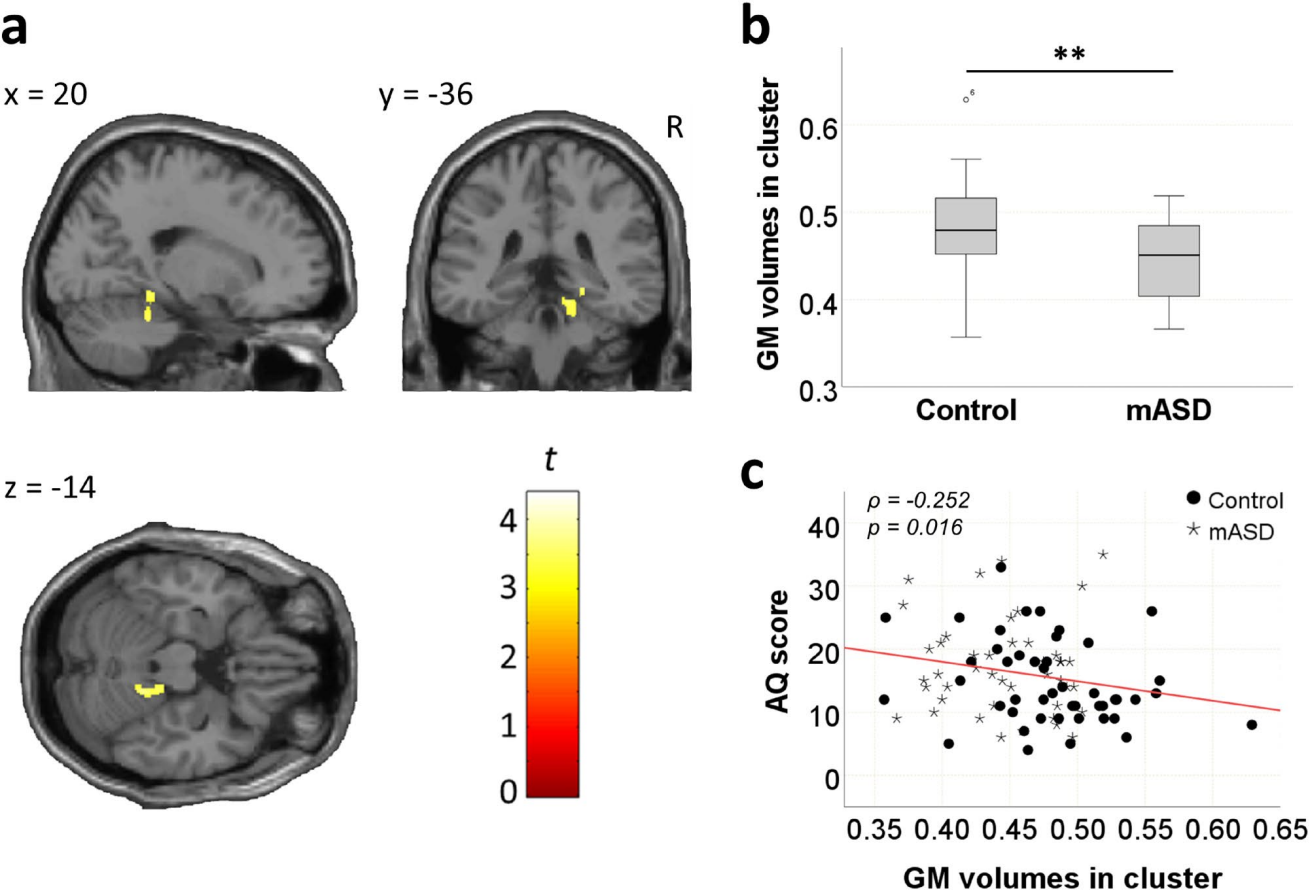
Grey matter volume differences between the mASD and control groups in the second cluster. (**a**) The second significant cluster was observed in the right cerebellum and parahippocampal gyrus. (**b**) Grey matter volume of the cluster was significantly different between the mASD and control groups (*t*(89) = 3.528*, P* = 0.001). (**c**) Scatter plot showing negative correlation between the grey matter extraction of the cluster and AQ scores for all subjects. The grey matter volume of the cluster was negatively correlated with AQ scores (ρ =  − 0.252,* P* = 0.016). **P < 0.01. *GM* grey matter, *mASD* mothers of individuals with autism spectrum disorder, *AQ* autism spectrum quotient.

We observed the first significant cluster in the right MTG (88.42%) and OUTSIDE (9.60%) regions (Table 2). The anatomical label “OUTSIDE” indicates that this part of the region is outside the brain parcellation. This cluster comprised the posterior part of the MTG and stretched into the parietal side. According to a previous study on brain anatomy of the TPJ^46^, the parietal part of this cluster can be defined as the brain region at or outside the posterior and inferior edge of the TPJ. The mASD group had smaller GM volume in this cluster compared with the control group (*t*(89) = 4.330*, P* = 0.000) (Fig. 2a,b). Furthermore, the mASD group also had smaller GM volume in the second cluster (*t*(89) = 3.528, *P* = 0.001) (Fig. 3a,b). The second cluster was labelled as the right cerebellum (85.71%) and the parahippocampal gyrus (9.07%) (Table 2). There were no significant increases in GM volume in the mASD group relative to the control group.

**Table 2:** Brain regions with significant volume differences between the mASD and control groups.

Cluster size	*q*FDR	Peak MNI coordinates in mm				Brain regions (AAL)
		*x*		*y*	*z*	
**mASD < control**						
354	0.014	42		− 69	8	Temporal_Mid_R (88.42%)
						OUTSIDE (9.60%)
364	0.014	18		− 41	− 24	Cerebellum_4_5_R (54.67%)
						Cerebellum_3_R (31.04%)
						ParaHippocampal_R (9.07%)
**mASD > control**						
None						

Only clusters with *q*FDR < 0.05 and their maximum peak voxels are presented; only AAL-defined regions comprising ≥ 5% of a cluster are listed. The “OUTSIDE” anatomical label indicates a part of the region outside the parcellation.

*AAL* automated anatomical labelling, *MNI* Montreal Neurological Institute, *mASD* mothers of individuals with autism spectrum disorder, *qFDR* false discovery rate *q*-value.

We also identified correlations between the significant GM volume clusters and behavioural traits, using Spearman’s rho test. AQ scores were negatively correlated with the second cluster (ρ =  − 0.252,* P* = 0.016) (Fig. 3c). However, they were not correlated with the first cluster (*P* > 0.05). In contrast, SQ scores were negatively correlated with the first cluster (ρ =  − 0.249,* P* = 0.017) (Fig. 2c), but were not correlated with the second cluster (*P* > 0.05).

## Discussion

ASD is one of the most heritable major neurodevelopmental disorders. Unaffected relatives of individuals with ASD, and especially the parents of individuals with ASD, have been widely reported to have subclinical forms of behavioural and neurobiological patterns that are characteristic of ASD. This feature of the parents of individuals with ASD has been termed the BAP. However, the brain structural features of parents of individuals with ASD are poorly understood and findings remain controversial.

In the present study, we observed the phenomenon of BAP through the subthreshold autistic-like behavioural features of mASD. We found qualitative differences in AQ and SQ scores between the mASD and control groups, although these were not significant when corrected for multiple comparisons. These findings suggest that parents of children with ASD have subclinical elevations in ASD traits.

To investigate the brain structural features of parents of individuals with ASD, we used VBM to examine brain GM volume in 45 unaffected mASD and 46 age-, sex-, and handedness-matched controls. We identified smaller GM volume in the MTG, TPJ, cerebellum, and parahippocampal gyrus in the mASD group. Moreover, we found that the volume of the significant MTG and TPJ cluster was negatively correlated with SQ scores, which assess the autistic drive to analyse or construct systems. Additionally, the volume of the significant cerebellum and parahippocampal gyrus cluster was negatively correlated with AQ scores, which measure autistic traits.

The MTG is associated with language, emotion, and social cognition (i.e. theory of mind or mentalization)^47–50^ and may be related to dysfunctions in mentalization and social processing in ASD^51–56^. Structural and functional imaging studies have shown that the MTG is atypical in individuals with ASD, but findings remain controversial. A previous study reported that an ASD group showed decreased GM volume in the MTG, similar to our findings that the mASD group had smaller MTG volume^57^. In contrast, several previous meta-analytic studies found that an ASD group had increased GM volume in the MTG^58,59^. Other meta-analytic studies have reported both increased and decreased GM volume in the MTG in ASD participants^60,61^. Regarding brain connectivity, individuals with ASD have hypoconnectivity of the posterior part of the MTG, indicating an association between the MTG and social cognition^62,63^.

The TPJ is a multimodal association area that receives and integrates input from the thalamus and multiple sensory modalities. Abnormalities in the higher-order integration area have been associated with lack of a mentalizing ability (i.e. theory of mind)^64,65^. The TPJ may play an important role in mentalizing, and mentalizing deficits are considered a key characteristic of ASD^66–70^. Moreover, TPJ abnormalities are one of the most consistent findings of brain structure^71,72^ and function^67,73^ in ASD.

The MTG and TPJ have often been implicated as aspects of the social brain, along with the superior temporal sulcus, fusiform gyrus, amygdala, anterior cingulate cortex, medial prefrontal cortex, and inferior frontal gyrus^28,29,74,75^. Abnormalities in the social brain regions have been widely suggested to result in poor social interactions in individuals with ASD^30,31,73,76–78^.

In the present study, smaller GM volumes within the MTG and TPJ correlated with higher SQ scores. The SQ assesses the drive to systemize, analyse, control, and construct rule-based systems, and all these features are involved in ASD^37^. Systemizing characteristics may mean that an individual is more interested in objects and/or rule-based systems than in social communication^37,79^. We thus speculate that a stronger drive to systemize is correlated with a smaller volume in social brain areas such as the MTG and TPJ. We suggest that the atypical GM volume of these social parts of the brain (i.e. the MTG and TPJ) may represent a potential neuroendophenotype of ASD.

We also identified a smaller volume in the cerebellum and parahippocampal gyrus of the mASD group. Although the cerebellum accounts for only 10% of the brain’s volume, it contains over half of all brain neurons^80^. The cerebellum plays an integrative role in the brain and connects many brain regions^32,81–84^. The cerebellum receives sensory information and conveys outputs to influence motor function through the cerebello-thalamocortical loops^84–86^. The cerebello-thalamocortical loops also have interconnections with the cerebral cortices and contribute to cognitive processing, including visuospatial perception, auditory processing, executive functions, and language skills^81,83,84,86,87^. The cerebellum is also linked to the frontal cortex^88^ and is involved in high-order cognitive functions^82,83^. Together, these findings indicate that the cerebellum is associated not only with motor coordination, but also with various forms of cognitive processing^81,89,90^.

Cerebellar abnormalities have been reported in individuals with ASD from early life through to adulthood^33,34,91–94^. Several previous studies on brain structure in ASD have reported decreased GM volume in the cerebellum in ASD groups, similar to our findings for the mASD group^58,59^. However, some meta-analytic studies have found that ASD groups showed decreased as well as increased GM volume in the cerebellum^60,95^. Recently, cerebellar neuropathology has gained attention as a means of explaining the characteristics of ASD^32,87,96^. Cerebellar abnormalities may be related to some ASD characteristics, such as a high rate of motor dysfunction, atypical sensory responsiveness, and impaired communication^97–99^.

The parahippocampal gyrus is an important pathway to the hippocampus and mediates convergent neocortical information for memory representations^100^. Functional MRI studies have shown that the right anterior parahippocampal gyrus is involved in the interactions between memory and emotion^101^, and may contribute to involuntary reactions associated with contextual fear memory that result in avoidance behaviour^102^. Previous brain structure studies have reported that individuals with ASD have abnormal patterns in the parahippocampal gyrus^34,103,104^. Furthermore, a reduction in parahippocampal GM volume may be related to the tendency to ignore dangers in individuals with ASD^104^.

In the present study, smaller GM volumes within the cerebellum and parahippocampal gyrus were related to higher levels of autistic traits. The cerebellum and parahippocampal gyrus play a role in receiving information from the cerebral cortex, and contribute to influencing emotional processing and various types of cognitive processing. We speculate that abnormalities in these brain areas may result in an atypical mediating pattern for emotional and cognitive processing in ASD. The small GM volumes in these brain regions might be related to subclinical ASD behavioural features, and may reflect a heritable neurobiological feature of ASD.

Our findings did not replicate the results of previous structural MRI studies of parents of individuals with ASD. There have been three such studies and they have reported inconsistent findings^26,35,36^. Rojas et al. measured volume in the hippocampus and amygdala in ASD individuals, the parents of individuals with ASD, and control subjects using manual-tracing techniques^36^. Both ASD individuals and parents of individuals with ASD had increased left hippocampal volume compared with the control group. In contrast, Palmen et al. used a semi-automatic procedure to compare the volume of the total brain, cortical lobes, cerebral GM and WM, cerebellum, and ventricles in the parents of individuals with ASD and a control group^26^. They found no significant differences in brain volume between the two groups. Peterson et al. used a VBM-based approach to compare regional GM volumes between a relatively small number of subjects (23 parents of individuals with ASD and 23 control subjects; 15 mothers and 8 fathers per group)^35^. They reported that the parents of individuals with ASD had increased volumes of the superior temporal gyri, inferior and middle frontal gyri, superior parietal lobule, and anterior cingulate, as well as decreased left anterior cerebellar hemisphere volume. The discrepancies between these three studies may result from both different analytical methods (manual-tracing technique, semi-automatic technique, or VBM methods) and participant characteristics (the number or sex of subjects).

### Limitations

In the present study, we examined the GM volume in mothers of children with ASD, to exclude any potential confounding factors related to sex. To increase the generalizability of our findings, additional studies should include data from the fathers of children with ASD. In addition, further studies are required to investigate the relationship between brain imaging and gene expression, to provide more solid evidence for BAP neuroendophenotypes. We used AQ and SQ scores to assess autistic-like behavioural features of the participants. A study limitation is that these are self-report measures. We found that a considerable part of the first cluster was outside the anatomy parcellations (i.e. outside the brain) and the second cluster spans biologically distinct structures. These results may have been affected by motion, although we checked for noise and discarded noisy data using the Mahalanobis distance algorithm. It is necessary to confirm the present results using motion detection techniques in the future study.

## Conclusions

Previous findings on brain structure in individuals with ASD and their parents are controversial. In the present study, we calculated GM volume in mASD and control groups using the whole-brain VBM method. We found that compared with the control group, the mASD group had smaller GM volumes in the MTG, TPJ, cerebellum, and parahippocampal gyrus. Our findings provide evidence to clarify controversial findings regarding intermediate neurobiological patterns observed in relatives of individuals with ASD, which we hope will ultimately help elucidate the underlying neurobiology of ASD. We suggest that these brain regions represent heritable brain structural features of ASD.

## Data Availability

The datasets generated and/or analysed during the current study are not publicly available as they contain information that could compromise the privacy of research participants but are available from the corresponding author on reasonable request.
